# Gas6 Promotes Oligodendrogenesis and Myelination in the Adult Central Nervous System and After Lysolecithin-Induced Demyelination

**DOI:** 10.1177/1759091416668430

**Published:** 2016-09-14

**Authors:** Salman Goudarzi, Andrea Rivera, Arthur M. Butt, Sassan Hafizi

**Affiliations:** 1 School of Pharmacy and Biomedical Sciences, Institute of Biomedical and Biomolecular Science, University of Portsmouth, UK

**Keywords:** multiple sclerosis, myelin, oligodendrocyte, receptor tyrosine kinase, TAM receptors, Tyro3

## Abstract

A key aim of therapy for multiple sclerosis (MS) is to promote the regeneration of oligodendrocytes and remyelination in the central nervous system (CNS). The present study provides evidence that the vitamin K-dependent protein growth arrest specific 6 (Gas6) promotes such repair in *in vitro* cultures of mouse optic nerve and cerebellum. We first determined expression of Gas6 and TAM (Tyro3, Axl, Mer) receptors in the mouse CNS, with all three TAM receptors increasing in expression through postnatal development, reaching maximal levels in the adult. Treatment of cultured mouse optic nerves with Gas6 resulted in significant increases in oligodendrocyte numbers as well as expression of myelin basic protein (MBP). Gas6 stimulation also resulted in activation of STAT3 in optic nerves as well as downregulation of multiple genes involved in MS development, including matrix metalloproteinase-9 (*MMP9*), which may decrease the integrity of the blood–brain barrier and is found upregulated in MS lesions. The cytoprotective effects of Gas6 were examined in *in vitro* mouse cerebellar slice cultures, where lysolecithin was used to induce demyelination. Cotreatment of cerebellar slices with Gas6 significantly attenuated demyelination as determined by MBP immunostaining, and Gas6 activated Tyro3 receptor through its phosphorylation. In conclusion, these results demonstrate that Gas6/TAM signaling stimulates the generation of oligodendrocytes and increased myelin production via Tyro3 receptor in the adult CNS, including repair after demyelinating injury. Furthermore, the effects of Gas6 on STAT3 signaling and matrix *MMP9* downregulation indicate potential glial cell repair and immunoregulatory roles for Gas6, indicating that Gas6-TAM signaling could be a potential therapeutic target in MS and other neuropathologies.

## Introduction

Multiple sclerosis (MS) is an autoimmune disease of the central nervous system (CNS) that involves destruction of oligodendrocytes and of the myelin sheath they produce around axons. MS features an inflammatory response that causes gradual demyelination through formation of plaques or lesions in the CNS white matter, leading to disruption in nerve impulse transmission (Saxena et al., 2011). Remyelination occurs in MS by the recruitment of oligodendrocyte progenitor cells (OPCs) to the lesion site and their subsequent differentiation into myelinating oligodendrocytes, under the control of multiple extracellular and axon-derived factors (Wolswijk, 2002). However, remyelination by OPCs ultimately fails, due in part to their differentiation being suppressed by inhibitory factors and the lack of a supportive cellular environment (Franklin, 2002; Kuhlmann et al., 2008). Hence, there is a need for a greater understanding of the factors regulating OPC differentiation and the development of novel targets for promoting remyelination.

Growth arrest specific 6 (Gas6) is a vitamin K-dependent protein that is a ligand for the TAM (Tyro3, Axl, Mer) family of receptor tyrosine kinases (RTKs). The Gas6–TAM interaction is involved in a number of cellular processes including regulating cell survival, the immune system, inflammation, proliferation, migration, and removal of apoptotic cells and debris (Lu and Lemke, 2001; Hafizi and Dahlbäck, 2006a; Weinger et al., 2011), all of which are processes involved in MS. The TAMs appear to play a significant role in immune system regulation and macrophage activation, as TAM triple knockout mice feature a severe lymphoproliferative disorder accompanied by broad-spectrum autoimmunity (Lu and Lemke, 2001; Lemke and Lu, 2003). Gas6-TAM signaling also plays a role in oligodendrocyte survival (Shankar et al., 2003) and has been shown to enhance remyelination in a cuprizone-induced demyelination model (Binder et al., 2011). These observations together implicate Gas6 as a natural molecule with potential therapeutic properties in MS, through its ability to promote oligodendrocyte survival and maturation, concomitant to its suppression of the innate immune response. In the present study, we characterize the expression of TAM receptors in the mouse CNS and demonstrate that Gas6 promotes oligodendrogenesis directly via Tyro3 receptor, and consequently myelination after lysolecithin-induced demyelination *in vitro*.

## Materials and Methods

### Animals

C57/BL6 mice aged postnatal day 7 (P7), P14, and adults were used for developmental studies, and 2-month-old adults were used for *ex vivo* experiments utilizing optic nerves and organotypic cerebellar slices cultures. Also, a transgenic mouse line was used in which green fluorescent protein (GFP) was expressed under control of the *SOX10* gene promoter (Stolt et al., 2006; Azim and Butt, 2011). All experiments involving animals were approved by University of Portsmouth Ethics Committee and also by the Home Office Animals Scientific Procedures Act (1986). Animals were killed humanely by cervical dislocation, and brains were removed rapidly and placed in ice-cold saline or fixative prior to experiments.

### Real-Time Quantitative PCR

Total RNA was isolated from human oligodendrocyte precursor cells (HOPC; 1600; ScienCell, CA), human astrocyte-cerebellar (HA-c; 1810; ScienCell), cultured optic nerves, and cortex and cerebellum of mice at different ages (P7, P14, and adult) using GeneJET RNA purification kit (Thermo Scientific, Loughborough, UK). The RNA was reverse transcribed into first-strand cDNA (NanoScript RT kit, Primerdesign, Southampton, UK) prior to use in real-time quantitative polymerase chain reaction (qPCR) analysis. Samples from human cell lines and from cortex and cerebellum of different ages were used to quantify gene expression, using specific primers and probes (Integrated DNA Technologies, IDT; Leuven, Belgium; Supplementary Table 1) and a qPCR master mix (FastStart Essential DNA Probes Master; Roche, Burgess Hill, UK). Also, cDNA from cultured optic nerves was analyzed for expression of 84 MS-related genes in a qPCR mini-array format (RT^2^ Profiler™ PCR Array Mouse Multiple Sclerosis; PAMM-125 Z; Qiagen, Hilden, Germany). The human and mouse samples were normalized to *GAPDH* and *Cdc40* as respective housekeeping genes for each species. The *Cdc40* gene was selected based on our own preliminary study of 12 reference genes (geNorm; Primerdesign Ltd), which revealed *Cdc40* to be among the most stable of mouse reference genes (not shown). The qPCR data from various mouse CNS tissues were analyzed based on the relative standard curve method as described earlier (Vouri et al., 2015). The qPCR amplification data from human samples and from optic nerves used in qPCR mini-array were analyzed based on the 2^−ΔΔCt^ method, where ΔCt is Ct_Target gene_ – Ct_housekeeping gene_ and ΔΔCt is ΔCt_Gas6_ – ΔCt_Mock_, and 2^−ΔΔCt^ shows the fold up- or downregulation, where values >1 are upregulated and <1 are downregulated (Livak and Schmittgen, 2001). The qPCR mouse mini-array contained 84 different genes involved in different aspects of MS, including myelination, T-cell activation and signaling, adaptive immunity, inflammation, and apoptosis (listed in Supplementary Table 2).

### Western Blot

Total protein extracts were obtained from fresh brain regions from mice at P7, P14, and adult ages, as well as from cultured optic nerves, using lysis buffer composed of 50 mM Tris-HCl, 150 mM NaCl, 1% Triton X-100, 0.5% NP-40, 1 mM EDTA, 10 mM Na_4_P_2_O_7_, pH 8.0. The extracts were loaded in equal total protein amounts on a 10% SDS-polyacrylamide gel, and proteins were separated by electrophoresis as described earlier (Goudarzi et al., 2013). Separated proteins were then transferred to a polyvinylidene fluoride membrane (Immobilon-P; Millipore, Watford, UK). Membranes were first blocked in 3% nonfat dry milk (for normal antibodies) or 3% bovine serum albumin (for phospho-specific antibodies) in 25 mM Tris, 150 mM NaCl, 0.05% Tween-20, pH 8.0, for 1 h at room temperature (RT), after which they were incubated at 4℃ overnight with primary antibodies. The primary antibodies and their dilutions were as follows: anti-Tyro3 (1:500, C-20), anti-Axl (1:500, C-20), anti-GAPDH (1:500, V-18; Santa Cruz Biotechnology, Santa Cruz, CA), anti-actin (1:10000, A2066; Sigma-Aldrich, Poole, UK), anti-phospho-STAT3 (1:500; Cell Signaling Technology, Leiden, The Netherlands), anti-phospho-Tyro3 (“anti-phospho-MER/SKY”; 1:1000, Phospho-Tyr749/681; Abbexa, Cambridge, UK), and anti-myelin basic protein (MBP, 1:500, MAB386; Millipore, Darmstadt, Germany). Postincubation washing of the membrane (25 mM Tris-HCl, 150 mM NaCl, 0.05% Tween-20, pH 8.0) was followed by 1 h incubation with a horseradish peroxidase-conjugated secondary antibody (1:5000; Promega, Madison, USA; Dako, Denmark) at RT. Chemiluminescence detection reagent (Luminata Forte Western HRP Substrate, Millipore) was used to generate the signal, and bands were visualized using a CCD-based digital gel imaging system (Bio-Rad ChemiDoc™ MP Imaging System, Hemel Hempstead, UK). The intensity of each band was quantified by densitometry using *ImageJ* software (Schneider et al., 2012), and query protein band intensities were normalized against those of GAPDH or actin protein in each sample.

### Optic Nerve Culture

Mouse optic nerves were isolated and established in culture for several days as described earlier (Azim and Butt, 2011). Briefly, 2-month-old transgenic Sox10-GFP or wild-type mice were killed by cervical dislocation, and optic nerves were removed while still attached to the eyeball and immediately placed in ice-cold artificial cerebrospinal fluid. The tissue was placed onto semiporous membrane inserts (0.4 µm; Millipore) and covered with 2 ml culture medium containing 50% Opti-MEM™, 25% horse serum, 25% Hanks Balanced Salt Solution (Gibco Invitrogen, Paisley, UK), supplemented with 25 mM D-glucose (Sigma), and antibiotics (Penicillin G sodium 10,000 U/ml, streptomycin sulfate 1,000 µg/ml; Gibco Invitrogen) diluted to 1:500, in a six-well culture plate. For experiments, recombinant Gas6 protein (diluted in cell culture medium containing Dulbecco’s Modified Eagle Medium, supplemented with 10% fetal calf serum, 100 U/ml penicillin, 100 µg/ml streptomycin, and 2 mM L-glutamine; Lonza, Slough, UK) was added directly to the culture medium at 400 ng/ml final concentration. In some experiments, Gas6 or Axl inhibitors were added, including the Gas6 antagonists soluble Axl/Fc as ligand quencher (R&D Systems, Minneapolis, MN) and warfarin-Gas6 (produced in-house) (both previously used by us and not toxic; Stenhoff et al., 2004), and the Axl-specific small molecule inhibitor BGB324 (Stratech Scientific, Newmarket, UK), which was used at a concentration of 1 µM as previously reported by us as effective in culture (Vouri et al., 2015). The cultures were incubated for 3 days, or for 3 h in signaling activation experiments. Cell culture medium alone was used as mock treatment, the same as the medium containing recombinant Gas6, as described earlier (Vouri et al., 2015). The culture medium and treatments were replenished on the second day of incubation. After 3 days, optic nerves were detached from the eye and the tissue processed for either Western blotting or qPCR, and samples from transgenic Sox10-GFP mice were fixed (4% paraformaldehyde, 15% picric acid) for 1 h at RT, prior to whole mounting on slides in mounting medium (Vectashield; Vector Labs, Peterborough, UK) and confocal microscopic examination of Sox10+ cells. All experiments were repeated with cultures prepared from different animals at different times.

### Organotypic Cerebellar Slice Culture

Sagittal sections of 300 µm thickness were made from brains of C56/BL6 mice at ages P8 to P12, using a tissue chopper (Campden Instruments, Loughborough, UK). The sections were placed onto semiporous membrane inserts (0.4 µm; Millipore) and covered with 1 ml culture medium composed of 50% Minimum Essential Medium with Glutamax-1, 18% Earle’s Balanced Salt Solution (EBSS), 5% EBSS + D-glucose, 1% penicillin-streptomycin, and 10% horse serum (Gibco Invitrogen; De Simoni and Yu, 2006). The slices were maintained on 0.4 µm semiporous membrane inserts in a six-well plate at 37℃ in a humidified atmosphere with 5% CO_2_, for a total of 7 days. After 3 days in culture, the slices were either treated with Gas6 for 3 h to investigate signaling mechanisms or in separate experiments to induce demyelination; on Day 3 of incubation, the medium was replaced with fresh media containing 1 mg/ml lysophosphatidylcholine (LPC, lysolecithin; Sigma) or methanol as vehicle. To investigate the effect of Gas6 on demyelination, 400 ng/ml of recombinant Gas6 in conditioned culture medium and mock medium as control were added to the same wells as the LPC treatments. All treatments with LPC were incubated further for 24 h, following which the medium was removed and the slices were incubated for another 3 days in fresh medium in the presence of freshly added Gas6 or mock treatments. Symmetrical cerebellar sections were used for direct comparisons between mock and Gas6 treatments. MBP immunofluorescent staining was then performed on the sections and analyzed by confocal microscopy. For quantification of myelination, the numbers of complete myelinated axons of length greater than 50 µm were counted for each treatment using *ImageJ* software. The values were converted to percentages and normalized against the values for vehicle-treated sections.

### Immunofluorescence Confocal Microscopy

Cell counts of Sox10-GFP-positive oligodendrocytes or OPCs in optic nerves were performed on images taken with a laser scanning confocal microscope (Zeiss LSM 710, Cambridge, UK). *z*-stack images were taken from each optic nerve with dimensions of 212.34 × 212.34 µm in the *x-* and *y-*plane and 70 µm in the *z-*plane at 10 µm intervals. The values of cell counts represent the mean (±*SEM*) percentage (%) of cells per field of view.

Cerebellar sections were fixed in 4% paraformaldehyde overnight, then blocked for 1 h in 20% normal horse serum diluted in TBS-TX (41.9 mM, Tris HCl; 8 mM Tris base; 154 mM NaCl; and 4.6 mM Triton-X). The sections were then incubated overnight with primary antibodies diluted in TBS-TX at the following dilutions: anti-MBP (1:300, MAB386; Millipore), anti-APC (5ug/ml, Ab-7; Millipore), anti-NeuN (1:250, MAB377; Millipore), and anti-glial fibrillary acidic protein (GFAP, 1:400, AB5541; Millipore). The next day, after washing with TBS-TX, the slices were incubated with the secondary antibodies for 2 h.

### Statistical Analysis

All statistical analyses were performed using the software IBM SPSS Statistics 20 or Prism 6 (GraphPad Inc, La Jolla, CA). Animals were randomly assigned to treatment groups. All results are expressed as mean ± *SEM*, with each experiment performed a minimum 3 times unless otherwise stated in the figure legend, using multiple replicates per treatments. Differences between multiple treatments in optic nerve cultures and organotypic cerebellar slice culture were compared using one-way analysis of variance followed by Bonferroni post hoc test, and comparison between two groups was carried out using unpaired Student *t* test. A *p* value of less than .05 was considered to be statistically significant.

## Results

### Expression of Gas6 and TAM Receptors in the Mouse CNS and in Human Glial Cells

**Figure 1.**
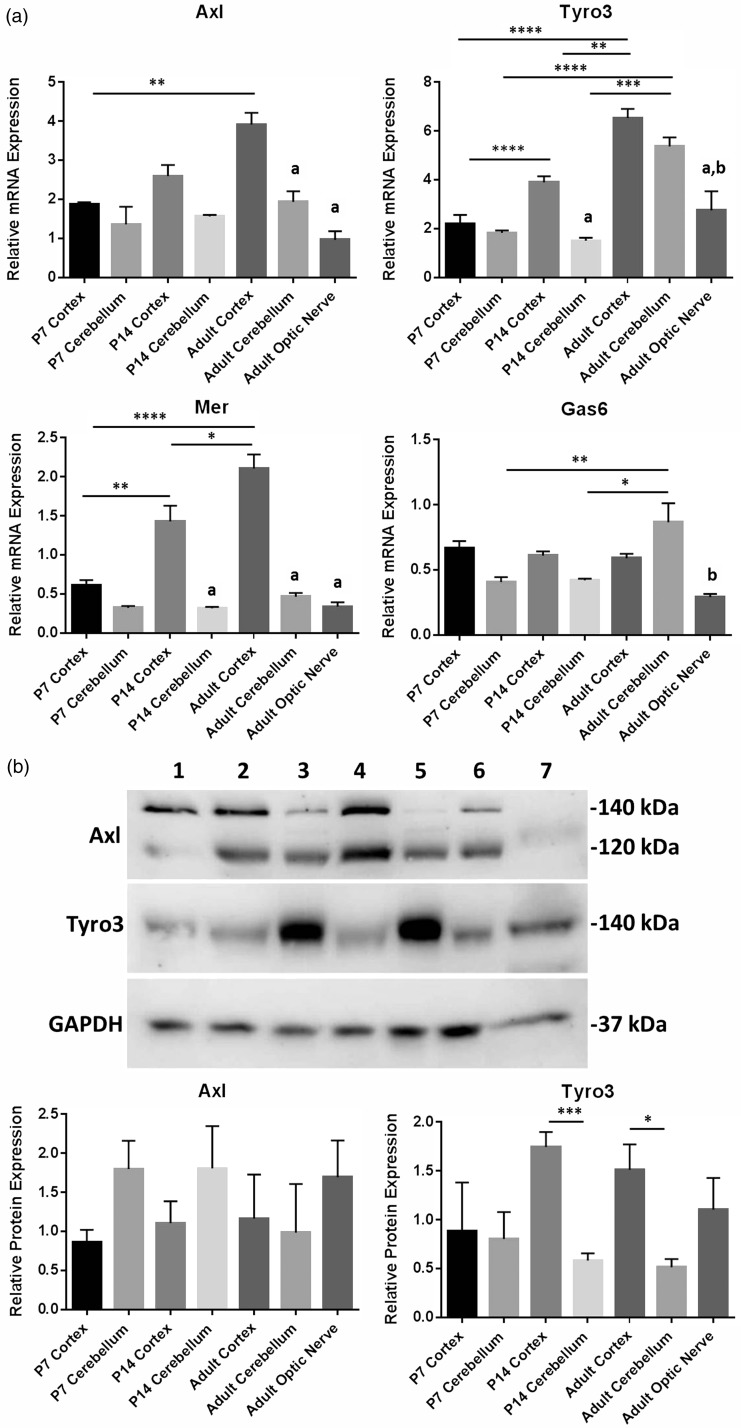
Expression of TAM receptors and Gas6 in different mouse brain regions and at different postnatal ages. (a) qPCR expression analysis of mRNA for Gas6 and TAM receptors. Values represent mean ± *SEM* of relative gene expression; *n* = 4 for all samples except for optic nerve (*n* = 3) and P14 cerebellum (*n* = 2). All samples were normalized against *Cdc40* as internal control; *p < .05, **p < .01, ***p < .001, ****p < .0001 for comparisons as indicated. Columns containing letter ***a*** are significant compared with cortex of the same age, and columns containing letter ***b*** are significant compared with cerebellum of the same age. (b) Representative Western blot of Axl, Tyro3, and GAPDH (loading control) proteins. Lanes correspond to the following: (1) P7 cortex, (2) P7 cerebellum, (3) P14 cortex, (4) P14 cerebellum, (5) adult cortex, (6) adult cerebellum, and (7) adult optic nerve. The histograms show the quantification of Axl and Tyro3 protein expression by densitometric analysis. Values represent mean ± *SEM* (*n* = 4 blots); *p < .05, #*p* = .056 between samples as indicated by lines. Columns containing letter ***a*** are significant compared with cortex of the same age, and columns containing letter ***b*** are significant compared with cerebellum of the same age.

TAM receptors are expressed in a wide variety of tissues and organs throughout the body and, while it is indicated that Tyro3 might be the main receptor in the CNS (Pierce and Keating, 2014), the expression profile of TAMs and Gas6 in the CNS remains unclear. To obtain a comprehensive picture of the whole TAM RTK subfamily and Gas6 in the mouse CNS, qPCR was performed on mRNA from different regions of the CNS and at different ages of postnatal development. TAM and Gas6 mRNA expression were detected in cortex and cerebellum of P7, P14, and adult mice and in adult optic nerve (Figure 1(a)). Furthermore, mRNA expression of all three TAMs increased significantly with age in the cortex but not in the cerebellum. This qPCR expression data are valid only for comparisons between samples per gene and not between samples for different genes. This is because the primer or probe sets for different genes will not have the same amplification dynamics and so the arbitrary relative expression values are valid only for a sample set for each gene.

In addition, Western blots showed clear expression of both Axl and Tyro3 proteins in all mouse brain regions and at all ages (Figure 1(b)), as well as in the adult optic nerve. However, Tyro3 protein expression was significantly higher in cortex versus cerebellum in P14 and adult, while the opposite was true of Axl expression (Figure 1(b)). The two bands seen in Axl Western blots are ∼120 kDa and ∼140 kDa forms of Axl due to different levels of glycosylation, reflecting different levels of maturation of the RTK.

**Figure 2.**
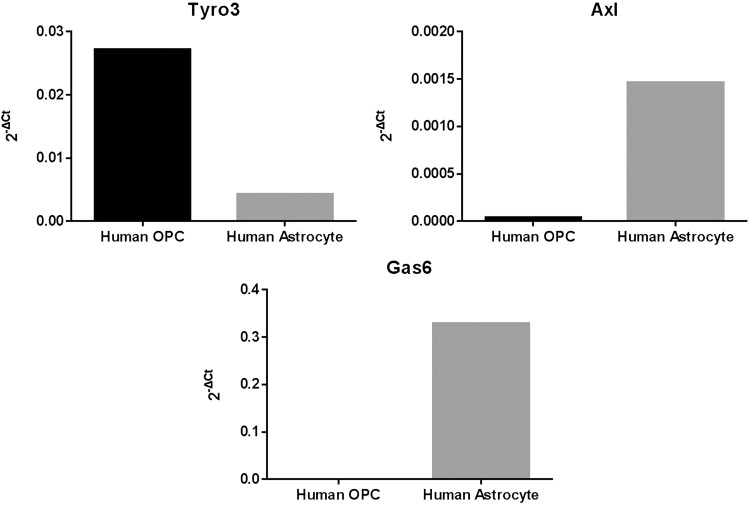
Expression of Tyro3, Axl, and Gas6 mRNA in human glial cell cultures. Extracted mRNA from human astrocytes and human oligodendrocyte precursors were analyzed by qPCR using specific primers for human Tyro3, Axl, and Gas6 genes. Results were analyzed based on 2^−ΔCt^ method.

The mRNA expression of Gas6, Tyro3, and Axl was also analyzed by qPCR in cultured HOPCs and HA-c, using the 2^−ΔCt^ method where ΔCt is Ct_target gene_ − Ct_internal control_ and 2^−ΔCt^ is the relative gene expression (Schmittgen and Livak, 2008). Tyro3 was expressed clearly in HOPC, whereas Axl was absent in this type of glial cells. In stark contrast, Gas6 was highly expressed in HA-c, while it was absent in HOPC (Figure 2).

### Gas6 Promotes Oligodendrogenesis in the Mouse Optic Nerve

**Figure 3.**
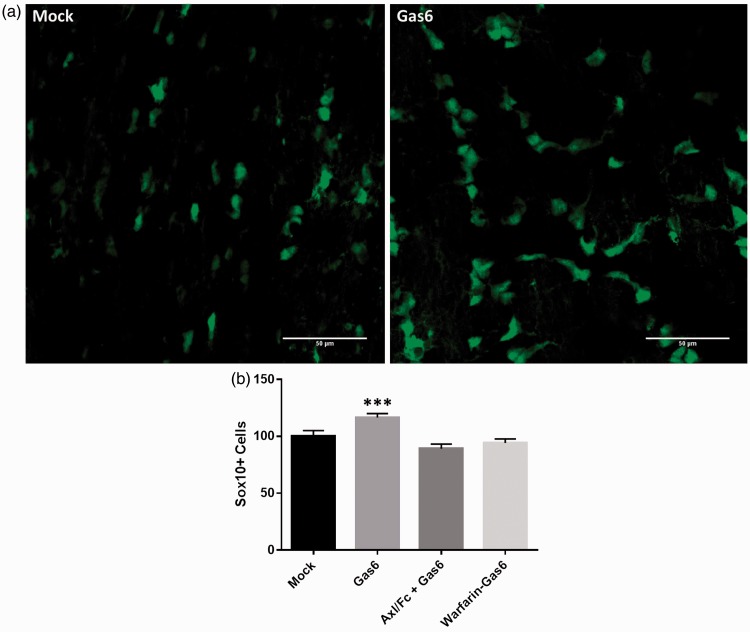
Effect of Gas6 on number of OPCs or oligodendrocytes in cultured mouse optic nerves. (a) Confocal images of optic nerves treated with mock medium (−Gas6) and Gas6, showing Sox10+ cells with green fluorescence. (b) Cell counts from images taken from optic nerves treated with Gas6 in the absence or presence of two Gas6 antagonists: soluble Axl receptor (Axl/Fc) and Warfarin-Gas6. Values represent mean ± *SEM* (control and Gas6, *n* = 8; Axl/Fc + Gas6, *n* = 2; Warfarin-Gas6, *n* = 4; *n* represents number of separate experiments); ***p < .001 versus control (*SEM* values are as follows: Mock = 5.0648, Gas6 = 3.5669, Axl/Fc + Gas6 = 4.0488, Warfarin-Gas6 = 3.4071, analysis of variance with Bonferroni correction).

To assess the effect of Gas6 on oligodendrogenesis, we used the optic nerve from adult Sox10-EGFP reporter mice, in which the reporter is expressed by myelinating oligodendrocytes, which make up the bulk of glia in the optic nerve, together with adult OPCs, which make up 3% to 5% of cells in the adult optic nerve (Butt et al., 2004). Optic nerves were isolated with retina intact and maintained in organotypic culture for 3 days, in control medium or medium containing Gas6. Samples also included Gas6 together with warfarin, which blocks the vitamin K-dependent γ-carboxylation of the protein posttranslation (Korshunov, 2012) and therefore produces Gas6 that is uncarboxylated and inactive, or the recombinant extracellular domain of Axl (Axl/Fc), which can act as a ligand antagonist by quenching the ligand before it binds to the receptor. After 3 days, optic nerves were fixed and whole mounted for confocal analysis and cell counts. The results show that Gas6 significantly increased the number of Sox10-GFP positive cells, and this effect was blocked by both Axl/Fc and warfarin (Figure 3; *p* < .001). These results were obtained from a number of experiments performed on different days, using separate cultures from different animals.

### Gas6 Enhances Myelination Following Lycolecithin-Induced Demyelination in Organotypic Cerebellar Slice Cultures

**Figure 4.**
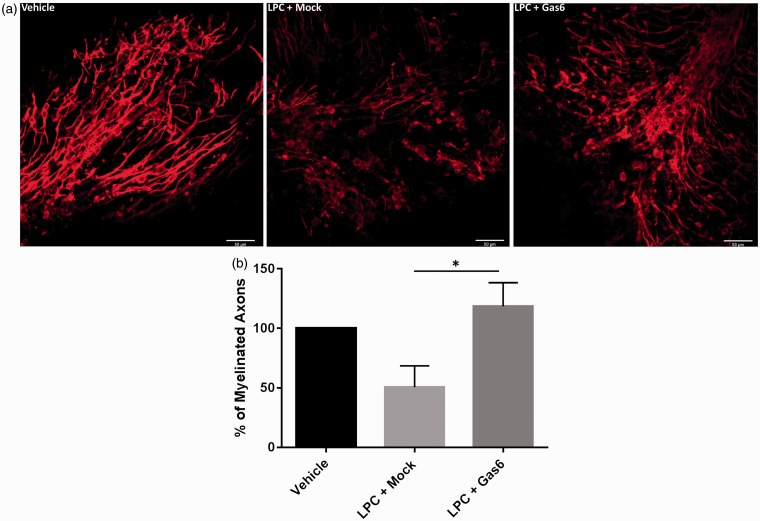
Gas6 significantly attenuates demyelination in a toxin-induced demyelination model. (a) Cerebellar slice cultures were treated with vehicle (methanol), LPC + Mock medium (−Gas6), and LPC + Gas6 and stained with MBP antibody. For each treatment within an experiment, one cerebellar slice was used, and one field of view per slice was analyzed. Images were taken from the top end of the lobules were the axons spread out. Scale bar = 50 µm. (b) Quantification of myelination through the number of MBP + axons with lengths greater than 50 µm. Values represent mean ± *SEM* (*n* = 6 experiments); *p < .05 for comparison indicated, analysis of variance with Bonferroni correction.

Having observed that Gas6 promotes oligodendrogenesis and myelination in the adult optic nerve, we next examined whether Gas6 could promotes remyelination after toxic injury, using lysolecithin (LPC) to cause destruction to the myelin in a cerebellar slice culture model, which also features spontaneous repair (Birgbauer et al., 2004; Zhang et al., 2011). Cerebellar slices were obtained from mice aged between P8 and P12 and cultured for 3 days to allow myelin formation to occur prior to adding LPC for 24 h, in normal medium or medium containing Gas6, followed by a further 3 days culture following LPC removal. Myelin loss was observed in slices treated with LPC, whereas demyelination was significantly decreased by approximately twofold in slice cultures coincubated with Gas6 (Figure 4; *p* < .05).

### Gas6 Activates STAT3 Signaling and Myelination in Optic Nerves

**Figure 5.**
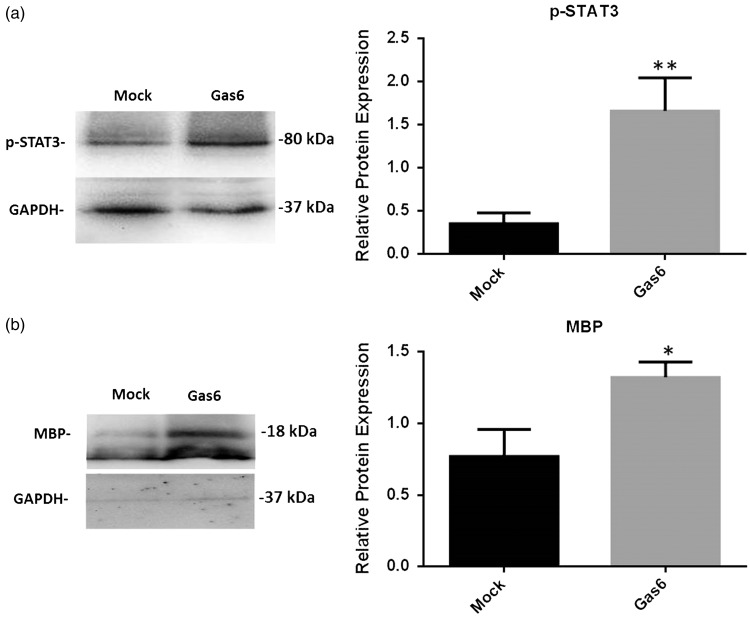
Effect of Gas6 on activation of intracellular STAT3 and myelination through MBP expression in cultured optic nerves. Representative Western blots are shown of proteins (a) pSTAT3 and (b) MBP in optic nerve lysates. The graphs accompanying each blot show the densitometric quantification of protein levels relative to GAPDH protein in those samples. Values represent mean ± *SEM* (*n* = 6 blots); *p < .05, **p < .01 versus control, Student *t* test. Gas6 significantly increased the phosphorylation of STAT3 as well as MBP in optic nerve cultures.

The JAK/STAT signaling pathway is a primary target of Gas6 signaling (Yanagita et al., 2001) and has been shown to promote oligodendrocyte regeneration and remyelination (Hesp et al., 2015), as well as being a promising therapeutic target in multiple animal models of MS (Liu et al., 2014). Therefore, we used Western blot to determine STAT3 activation through detecting levels of phosphorylated STAT3 (p-STAT3; Figure 5(a)) as well as levels of MBP as a measure of differentiation or myelination (Figure 5(b)). The protein extracts from cultured adult optic nerves were analyzed, and Western blot band intensities were quantified by densitometric analysis, normalizing against GAPDH protein (Figure 5). The results demonstrate that compared with controls, Gas6 stimulation (400 ng/ml) resulted in a significant increases in p-STAT3 protein (Figure 5(a); *p* < .01) as well as MBP, which was increased approximately twofold (Figure 5(b); *p* < .05). The results demonstrate that Gas6 activates STAT3 signaling and stimulates the molecular process of differentiation or myelination in the adult optic nerve.

### Gas6 Stimulates STAT3 Phosphorylation in Mature Oligodendrocytes and Astrocytes

**Figure 6.**
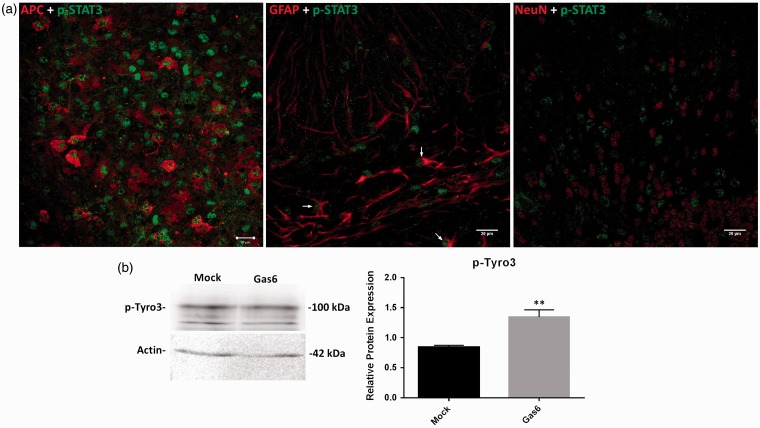
Gas6 activates STAT3 in mature oligodendrocytes and astrocytes and activates Tyro3 receptor after 3 h stimulation in culture. (a) Cerebellar slices were treated with mock or Gas6 medium for 3 h, and fixed sections were double stained for p-STAT3 and APC (mature oligodendrocyte marker), p-STAT3 and GFAP (astrocyte marker), p-STAT3 and NeuN (neuronal marker; scale bar = 20 µm). The stainings reveal that p-STAT3 is present in oligodendrocytes and astrocytes but not in neurons. The arrows point to cells that are both GFAP+ and p-STAT3+. (b) Representative Western blot of p-Tyro3 protein in optic nerve lysate. The graph shows the densitometric quantification of protein level relative to actin in the same sample. Values represent mean ± *SEM* (*n* = 5 blots); **p < .01 versus control, Student *t* test. Gas6 significantly increased the activation of Tyro3.

Having observed by Western blot that Gas6 stimulates STAT3 phosphorylation in cultured optic nerves, we then investigated the specific cell types in which this STAT3 activation occurred, through confocal microscopic analysis of cerebellar slice cultures. After 3 h culture incubation in the presence of Gas6, fixed cerebellar sections were double stained with p-STAT3 antibody together with antibodies for mature oligodendrocyte, astrocyte, and neuronal markers. The double immunostaining of Gas6-treated tissues showed p-STAT3 to be present predominantly in mature oligodendrocytes and also in some astrocytes but not in neurons (Figure 6(a) and Supplementary Figure S3). Our data indicate that treatment of cerebellar slices with Gas6 for only 3 h is enough for activating STAT3, and thus the effect of Gas6 on increased level of STAT3 phosphorylation is not due to increased level of STAT3 protein, which is a longer term process.

Having observed that oligodendrocytes express Tyro3, we attempted to determine whether Tyro3 was the TAM receptor that mediated the observed effects of Gas6. For this, we performed Western blots to investigate levels of phosphorylated (activated) Tyro3 (p-Tyro3) in cultured mouse optic nerves treated with Gas6 for 3 h. The Western blotting shows that compared with control, Gas6 stimulated a significant increase in p-Tyro3 protein levels in optic nerves, showing that Gas6 directly activates Tyro3 in the white matter (Figure 6(b)). The apparent discrepancy between the molecular weight of total Tyro3 (∼140 kDa) and p-Tyro3 (∼100 kDa) is due to the respective antibodies binding to different forms of Tyro3. The anti-Tyro3 antibody is highly selective for the fully mature Tyro3, whereas the anti-p-Tyro3 recognizes the smaller band species of Tyro3. We also performed these experiments in the presence of a specific small molecule inhibitor of Axl and BGB324 (Barcena et al., 2015; Vouri et al., 2015) and observed that blocking Axl did not affect the Gas6 effect on myelination, further indicating that the myelination effect occurs specifically via Tyro3 receptor (Supplementary Figure S1).

### Gas6 Alters MS-Related Gene Expression in Cultured Optic Nerves in a Promyelinating Direction

**Figure 7.**
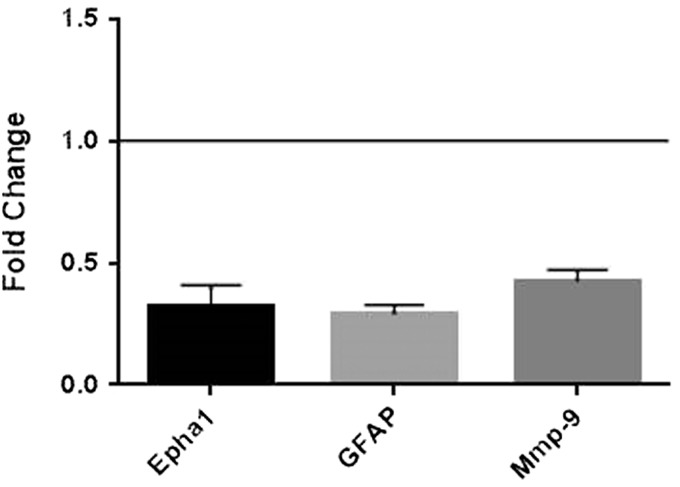
Effect of Gas6 on expression of selected MS-related genes in cultured mouse optic nerves. qRT-PCR was performed on extracts from mock and Gas6-treated optic nerve cultures, using specific primer or probe sets, and normalizing expression against the *GAPDH* gene. Treatment of optic nerves with Gas6 resulted in downregulation of *Epha1*, *GFAP*, and *MMP9* genes. Values represent mean ± *SEM* (*n* = 4 for qPCR experiments for all genes except for *Epha1* (*n* = 2)).

The effect of Gas6 on expression of 84 different MS-related genes was examined in the adult optic nerve, using the Mouse Multiple Sclerosis PCR array (Qiagen). Fifteen genes were identified as being significantly altered by Gas6 treatment compared with controls (Table 1), seven being upregulated ≥ twofold and eight downregulated ≥ twofold. Among those, some of the altered genes were selected for further individual analysis by qPCR, including the genes for matrix metalloproteinase-9 (*MMP9*), which is able to mediate blood–brain barrier (BBB) injury (Ravindran et al., 2011), the astrocyte marker GFAP (Scheller and Kirchhoff, 2009), and Epha1, which is an RTK (Owshalimpur and Kelley, 1999). The individual follow-up qPCR analyses confirmed that *MMP9*, *Gfap*, and *Epha1* genes were all significantly downregulated in the optic nerve following Gas6 treatment (Figure 7).

**Table 1.** Summary of Genes in Cultured Mouse Optic Nerves Altered by Gas6 Stimulation by ≥2-Fold.

Gene Symbol	ΔCt		Fold upregulation or downregulation	
	Gas6	Mock	ΔΔCt	2^−ΔΔCt^
*Ccl3*	3.06	4.18	−1.12	2.173469725
*Ccl7*	0.45	1.62	−1.17	2.250116969
*Cxcl11*	8.86	10.31	−1.45	2.732080514
*Fasl*	11.71	13.39	−1.68	3.20427951
*H2-Eb1*	12.12	14.07	−1.95	3.863745316
*Il13*	11.82	13.29	−1.47	2.770218936
*Il1b*	8.65	9.69	−1.04	2.056227653
*Ednra*	8.08	6.65	1.43	0.371131
*Epha1*	9.36	8.06	1.3	0.406126198
*GFAP*	1.06	−0.38	1.44	0.368567304
*Mag*	2.09	0.79	1.3	0.406126198
*MMP9*	6.43	5.41	1.02	0.493116352
*Nr2f1*	7.27	5.47	1.8	0.287174589
*Tubb4*	3.04	2.01	1.03	0.489710149
*Vcam1*	2.5	1.3	1.2	0.435275282

## Discussion

The three homologous TAM receptors and their common ligand Gas6 are expressed widely throughout the body (Hafizi and Dahlbäck, 2006a, 2006b). However, their expression profiles in the CNS, and their functional roles therein, remain to be comprehensively characterized. In the present study, we show that Gas6 and all three TAM receptors are expressed in the mouse brain at mRNA level, as well as Tyro3 and Axl protein, and that their expression increases during myelination and is highest in the adult cortex. In addition, we show that Gas6 promotes the generation of oligodendrocytes and myelin in the white matter of the adult mouse optic nerve and also attenuates myelin loss in the lysolecithin model of demyelination in cerebellar slice cultures. Furthermore, analysis of the molecular mechanisms of action of Gas6 demonstrates that it activates the Tyro3 receptor, intracellular STAT3 signaling, and the programming of a host of MS-related genes in a prorepair and myelinating direction. These results therefore indicate that Gas6 or TAM signaling is a potential target for stimulating repair mechanisms that are relevant to demyelination and MS.

All three TAM receptors were observed to be expressed throughout the brain tissue, although Tyro3 was expressed in more regions and at higher levels compared with Axl and Mer. This supports previous indications that Tyro3 is the primary TAM receptor mediating the actions of Gas6 in the CNS (Prieto et al., 2000). In addition, our data show that Tyro3 expression increases during development in the CNS. These observations are in line with previous time-course expression data, showing Tyro3 protein to be very low at embryonic stages while its expression begins to increase shortly after birth, dramatically increasing between postnatal days P3 and P15, reaching its peak level at P24, and remaining at that level thereafter in the adult (Prieto et al., 2000). Although most myelination occurs early in life, myelination carries on at least into late adolescence and, in some regions of the CNS, may increase throughout much of adult life (Emery, 2010). Therefore, the concomitant temporal profiles of myelination and Tyro3 expression in the developing mouse CNS suggest that Tyro3 plays a role in oligodendrocyte development or maturation and myelination. In the Axl blot, a double band was observed at both 120 kDa and 140 kDa, which are the well-known alternately glycosylated versions of Axl (the 140 kDa protein being most mature; O’Bryan et al., 1995). Our results showed clear expression of Axl protein in all brain regions and ages analyzed, with no significant differences in levels of expression among them.

We also conducted qPCR analysis for Tyro3, Axl, and Gas6 genes in separate pure cultures of human OPCs and astrocytes. Tyro3 was highly expressed in oligodendrocytes, in which Axl expression was absent. This key finding therefore suggests that Gas6 acts directly on OPCs or oligodendrocytes via the Tyro3 receptor to affect their fate and function. In contrast, Tyro3 expression was lower in astrocytes compared with oligodendrocytes but instead astrocytes showed a high level of Gas6 expression. This therefore suggests an indirect and supportive role for astrocytes in regulating the fate and function of oligodendrocytes, by providing the ligand through paracrine signaling. However, this interpretation of relative TAM expression comes with a certain degree of caution as the human isolated glial cell cultures were not only from different brain regions but were also derived from different brains and at different ages. Nevertheless, within a specific cell type, our data clearly show that Tyro3 is present in oligodendrocytes, while Axl is virtually absent in these cells. Furthermore, according to Figure 1(a), Mer mRNA was also observed in the CNS, although we did not characterize its expression pattern in glial cells, but according to previous reports, it is mainly expressed in microglia within the CNS and is involved in phagocytic activity of these cells (Fourgeaud et al., 2016). Axl has also been reported to be present in microglia, and no Tyro3 was observed in these cells (Fourgeaud et al., 2016). Also, it was shown that, in addition to Mer, Axl is also essential for the phagocytic activity and noninflammatory properties of microglia cells, including removal of cell debris (Fourgeaud et al., 2016).

Moreover, as we have only analyzed glial cells in this study, it is important to note that expression of Gas6 expression has been reported in different neuronal cells including Purkinje neurons, motor neurons of spinal cord, as well as large dorsal root ganglia neurons (Li et al., 1996; Prieto et al., 1999). Therefore, it is also possible that neurons may play a similar supportive role in regulating oligodendrocyte development and function, as astrocytes do.

As well as in the brain, qPCR analysis of adult mouse optic nerve demonstrated that Gas6 or TAM signaling components are also present in this tissue, with relative levels of expression indicated in the following order Tyro3 > Axl > Mer. Also, Gas6 stimulation of optic nerve from Sox10-EGFP mice further confirmed the presence and activity of TAM receptors in CNS white matter, through which they promote the generation of oligodendrocyte lineage cells. The Sox10-EGFP reporter identifies both oligodendrocytes and OPCs, since Sox10 marks all stages of oligodendrocyte development (Stolt et al., 2006); but in the adult, OPCs are continuously generating new oligodendrocytes and are the likely targets of Gas6 in our study (Gonzalez-Pereza and Alvarez-Buyllab, 2011). In addition to oligodendrogenesis, Gas6 stimulated increases the levels of MBP protein in the adult optic nerve, which indicates that Gas6 also promotes myelination in mature oligodendrocytes (Mi et al., 2011). Therefore, these observations together demonstrate that Gas6 boosts both the number of OPCs as well as mature myelinating oligodendrocytes. In this regard, it is worthwhile considering protein S, a protein homologous to Gas6 that is also a TAM receptor ligand. However, its expression appears restricted in the CNS, with low levels of protein S mRNA observed in locus coeruleus and high levels of mRNA in the choroid plexus (Prieto et al., 1999). Furthermore, the expression of protein S appears to be primarily neuronal, having been detected in the cortex, hippocampal pyramidal neurons, and granule neurons of the dentate gyrus of the rabbit brain (He et al., 1995; Prieto et al., 2007).

Gas6 stimulation also led to a decreased demyelination in a cerebellar slice culture model of demyelination by lysolecithin (Birgbauer et al., 2004), which is one of the most commonly used toxins to induce focal demyelination in different parts of the brain (van der Star et al., 2012). Our results show that treatment with Gas6 significantly attenuated the extent of demyelination that continues for days after exposure to lysolecithin (Birgbauer et al., 2004). This is consistent with studies in the cuprizone model of demyelination, which showed systemic administration of Gas6 to enhance remyelination (Tsiperson et al., 2010), while Gas6 ablation decreased the number of myelinating oligodendrocytes and delayed recovery (Binder et al., 2011). Together, these studies demonstrate that Gas6 or TAM signaling plays a major role in oligodendrocyte generation and myelination in the adult CNS. However, our experiment was focused on the protective effect of Gas6 during demyelinating injury, whereas it is also important to investigate the acceleratory effect of Gas6 on remyelination after damage. Thus far, we did not observe an enhanced remyelination when Gas6 was added 3 days after LPC damage (Supplementary Figure S2). However, further detailed time-course studies are warranted to explore the time window during which Gas6 can be effective as an enhancer of remyelination post damage. Furthermore, although Gas6 increases MBP and blocks demyelination in culture, it is also worthwhile to measure the axon diameter or g-ratio by electron microscopy to determine specifically the location of myelination as being around axons, and hence contributing to increased axon thickness. Also, although this study was focused on the nonmicroglial response to Gas6, it is nevertheless possible, considering the phagocytosis-mediating role of Mer (Chen et al., 1997), that Gas6 could also activate Mer in microglia in our culture model; this requires further investigation.

In addition to the ligand Gas6, the role of Axl receptor has also investigated by other research groups in demyelination models. In a study using the cuprizone model of demyelination, Axl knockout mice showed no significant differences to wild-type mice in axon diameter, myelin thickness, and g-ratio of myelin-containing axons (Hoehn et al., 2008). This therefore suggests that Axl is not the main receptor involved in remyelination or that loss of signaling through all three TAM receptors is more harmful than loss of signaling through a single TAM receptor. This is consistent with our observations, which point toward the prominence of Tyro3 in oligodendrocyte biology and myelination, reinforced by its unique mRNA expression in oligodendrocytes.

As our expression analysis indicated Tyro3 as the most prominent TAM receptor in the CNS, at least as concerns oligodendrocytes, we therefore hypothesized that the Gas6 effects on OPC proliferation, oligodendrocyte maturation, and myelination are likely occurring via a direct activation of Tyro3 in these cell populations. We therefore utilized our optic nerve culture model to also investigate the relatively acute activation of Tyro3 in response to Gas6 stimulation. Thus, Gas6 stimulated a higher activation state of Tyro3 after 3 h in the optic nerve, as observed by increased p-Tyro3 protein levels. This observation is supported by a recent study in the peripheral nervous system that showed Tyro3 to be an important modulator of myelination by Schwann cells (Miyamoto et al., 2015).

We also observed that the transcriptional regulator protein STAT3 was mobilized by Gas6 stimulation using both of our *in vitro* culture models. Gas6 increased levels of phosphorylated STAT3 protein in the optic nerve, and using the slices for microscopy, we were able to localize the pSTAT3 to oligodendrocytes as well as to a lesser extent astrocytes. Gas6 has previously been reported to act via STAT3 to promote proliferation of mesangial cells (Yanagita et al., 2001). This is consistent with our current observations of Gas6-induced activation of STAT3 and proliferation of OPCs or oligodendrocyte in the optic nerve. STAT3 controls a large number of genes that are involved in apoptosis, cell migration, cell cycle regulation, and angiogenesis as well as modulation of immune suppressive factors (Ferguson et al., 2015). Once STAT3 becomes activated via phosphorylation, it translocates to the nucleus and binds to specific DNA sequences to activate the expression of target genes that regulate proliferation and differentiation (Qi and Yang, 2014). There is also evidence that shows STAT3 activation is potentially important during neuroinflammation by protecting myelin development (Nobuta et al., 2012). Although neurons also express Tyro3 (Schulz et al., 1995), we found Gas6 to induce STAT3 activation predominantly in OPCs or oligodendrocytes, as would be expected for differentiating or proliferating cells (Fukada et al., 1996). Therefore, STAT3 appears to be a key intracellular mediator of Gas6 signaling in oligodendrocytes, most likely via Tyro3, to affect their development and the subsequent myelination of axons.

From a mini-array screen, we also observed Gas6 to induce significant changes in expression of multiple genes relevant to glial biology and MS in the cultured optic nerve, including downregulation of *MMP9*, *Epha1*, and *GFAP* genes. GFAP is an astrocyte-specific protein and is used as a marker of reactive astrogliosis (Beckerman et al., 2015), suggesting that Gas6 may modulate astroglial responses in the optic nerve. MMP9 is a proteolytic enzyme that degrades type IV collagen, laminin, and fibronectin, all of which are major components of the basement membrane of the BBB (Zheng et al., 2014). During a relapse course of MS, active B cells increase the expression and secretion of MMP9 protein, ultimately resulting in digestion of myelin as well as neurological disability (Aung et al., 2015). Furthermore, it has been shown that MMP9 can be secreted by OPCs that have proliferated at sites of demyelination in the CNS, which results in opening of the BBB and the subsequent secondary cascades of cerebrovascular injury and demyelination (Seo et al., 2013). Moreover, disruption of the BBB has been considered as one of the initial steps in the development of disease in MS patients (Alvarez et al., 2011). Also, the role of MMP9, at least produced by active B lymphocytes, in the disruption of the BBB and degradation of MBP protein is well recognized (Aung et al., 2015). Thus, our observation of Gas6 causing a twofold downregulation of the *MMP9* gene suggests that Gas6 may help maintain BBB integrity and hence inhibit immune cell infiltration into the CNS during the course of MS; this is a focus of investigation in subsequent studies.

In conclusion, this study shows that components of Gas6 or TAM signaling are present throughout the adult CNS, and that, specifically, Tyro3 is present on OPCs or oligodendrocytes and mediates their development and function in response to the ligand Gas6, while astrocytes are the main source of the Gas6. Gas6 promotes the development and maturation of oligodendrocytes from OPCs and myelination in the adult CNS, as well as stimulating remyelination after toxic injury by lysolecithin. In addition, we show that the effects of Gas6 involve regulation of a set of genes that, through their coordinated upregulation or suppression, push the glial cell developmental pathway in a promyelinating direction. These results support a prominent role for Gas6 in promoting CNS repair after demyelination, and thus an attractive consideration for novel therapeutic approaches for MS.

## Summary

We detected regional and age-dependent expression of TAM family receptors in the mouse CNS. Their common ligand Gas6 boosted mature oligodendrocyte numbers, stimulated myelination, and dampened toxin-induced demyelination. Gas6 acted via Tyro3 receptor to activate intracellular STAT3 signaling in oligodendrocytes.

## Supplementary Material

Supplementary material

## Supplementary Material

Supplementary material

## References

[bibr1-1759091416668430] Alvarez J. I. Cayrol R. Prat A. (2011) Disruption of central nervous system barriers in multiple sclerosis. Biochimica et Biophysica Acta 1812: 252–264. 20619340 10.1016/j.bbadis.2010.06.017

[bibr2-1759091416668430] Aung L. L. Mouradian M. M. Dhib-Jalbut S. Balashov K. E. (2015) MMP-9 expression is increased in B lymphocytes during multiple sclerosis exacerbation and is regulated by microRNA-320a. Journal of Neuroimmunology 278: 185–189. 25468268 10.1016/j.jneuroim.2014.11.004 PMC4297694

[bibr3-1759091416668430] Azim K. Butt A. M. (2011) GSK3beta negatively regulates oligodendrocyte differentiation and myelination in vivo. Glia 59: 540–553. 21319221 10.1002/glia.21122

[bibr4-1759091416668430] Barcena C. Stefanovic M. Tutusaus A. Joannas L. Menendez A. Garcia-Ruiz C. Morales A. (2015) Gas6/Axl pathway is activated in chronic liver disease and its targeting reduces fibrosis via hepatic stellate cell inactivation. Journal of Hepatology 63: 670–678. 25908269 10.1016/j.jhep.2015.04.013 PMC4543529

[bibr5-1759091416668430] Beckerman S. R. Jimenez J. E. Shi Y. Al-Ali H. Bixby J. L. Lemmon V. P. (2015) Phenotypic assays to identify agents that induce reactive gliosis: A counter-screen to prioritize compounds for preclinical animal studies. Assay and Drug Development Technologies 13: 377–388. 26230074 10.1089/adt.2015.654 PMC4555645

[bibr6-1759091416668430] Binder M. D. Xiao J. Kemper D. Ma G. Z. Murray S. S. Kilpatrick T. J. (2011) Gas6 increases myelination by oligodendrocytes and its deficiency delays recovery following cuprizone-induced demyelination. PLoS One 6: e17727. 21423702 10.1371/journal.pone.0017727 PMC3053381

[bibr7-1759091416668430] Birgbauer E. Rao T. S. Webb M. (2004) Lysolecithin induces demyelination in vitro in a cerebellar slice culture system. Journal of Neuroscience Research 78: 157–166. 15378614 10.1002/jnr.20248

[bibr8-1759091416668430] Butt A. M. Pugh M. Hubbard P. James G. (2004) Functions of optic nerve glia: Axoglial signalling in physiology and pathology. Eye (Lond) 18: 1110–1121. 15534596 10.1038/sj.eye.6701595

[bibr9-1759091416668430] Chen J. Carey K. Godowski P. J. (1997) Identification of Gas6 as a ligand for Mer, a neural cell adhesion molecule related receptor tyrosine kinase implicated in cellular transformation. Oncogene 14: 2033–2039. 9160883 10.1038/sj.onc.1201039

[bibr10-1759091416668430] De Simoni A. Yu L. M. (2006) Preparation of organotypic hippocampal slice cultures: Interface method. Nature Protocols 1: 1439–1445. 17406432 10.1038/nprot.2006.228

[bibr11-1759091416668430] Emery B. (2010) Regulation of oligodendrocyte differentiation and myelination. Science 330: 779–782. 21051629 10.1126/science.1190927

[bibr12-1759091416668430] Ferguson S. D. Srinivasan V. M. Heimberger A. B. (2015) The role of STAT3 in tumor-mediated immune suppression. Journal of Neuro-oncology 123: 385–394. 25700834 10.1007/s11060-015-1731-3

[bibr13-1759091416668430] Fourgeaud L. Traves P. G. Tufail Y. Leal-Bailey H. Lew E. D. Burrola P. G. Lemke G. (2016) TAM receptors regulate multiple features of microglial physiology. Nature 532: 240–244. 27049947 10.1038/nature17630 PMC5358512

[bibr14-1759091416668430] Franklin R. J. M. (2002) Why does remyelination fail in multiple sclerosis? Nature Reviews 3: 9. 10.1038/nrn917 12209119

[bibr15-1759091416668430] Fukada T. Hibi M. Yamanaka Y. Takahashi-Tezuka M. Fujitani Y. Yamaguchi T. Hirano T. (1996) Two signals are necessary for cell proliferation induced by a cytokine receptor gp130: Involvement of STAT3 in anti-apoptosis. Immunity 5: 449–460. 8934572 10.1016/s1074-7613(00)80501-4

[bibr16-1759091416668430] Gonzalez-Pereza O. Alvarez-Buyllab A. (2011) Oligodendrogenesis in the subventricular zone and the role of epidermal growth factor. Brain Research Reviews 67: 9. 10.1016/j.brainresrev.2011.01.001 PMC3109119 21236296

[bibr17-1759091416668430] Goudarzi S. Smith L. J. Schutz S. Hafizi S. (2013) Interaction of DISC1 with the PTB domain of Tensin2. Cellular and Molecular Life Sciences: CMLS 70: 1663–1672. 23233134 10.1007/s00018-012-1228-6 PMC11113815

[bibr18-1759091416668430] Hafizi S. Dahlbäck B. (2006a) Gas6 and protein S. Vitamin K-dependent ligands for the Axl receptor tyrosine kinase subfamily. FEBS Journal 273: 5231–5244. 17064312 10.1111/j.1742-4658.2006.05529.x

[bibr19-1759091416668430] Hafizi S. Dahlbäck B. (2006b) Signalling and functional diversity within the Axl subfamily of receptor tyrosine kinases. Cytokine Growth Factor Reviews 17: 295–304. 16737840 10.1016/j.cytogfr.2006.04.004

[bibr20-1759091416668430] He X. Shen L. Bjartell A. Dahlback B. (1995) The gene encoding vitamin K-dependent anticoagulant protein S is expressed in multiple rabbit organs as demonstrated by northern blotting, in situ hybridization, and immunohistochemistry. The Journal of Histochemistry and Cytochemistry: Official Journal of the Histochemistry Society 43: 85–96. 7822769 10.1177/43.1.7822769

[bibr21-1759091416668430] Hesp Z. C. Goldstein E. A. Miranda C. J. Kaspar B. K. McTigue D. M. (2015) Chronic oligodendrogenesis and remyelination after spinal cord injury in mice and rats. The Journal of Neuroscience: The Official Journal of the Society for Neuroscience 35: 1274–1290. 25609641 10.1523/JNEUROSCI.2568-14.2015 PMC4300327

[bibr22-1759091416668430] Hoehn H. J. Kress Y. Sohn A. Brosnan C. F. Bourdon S. Shafit-Zagardo B. (2008) Axl-/- mice have delayed recovery and prolonged axonal damage following cuprizone toxicity. Brain Research 1240: 1–11. 18804096 10.1016/j.brainres.2008.08.076

[bibr23-1759091416668430] Korshunov V. A. (2012) Axl-dependent signalling: A clinical update. Clinical Science 122: 361–368. 22187964 10.1042/CS20110411 PMC3609429

[bibr24-1759091416668430] Kuhlmann T. Miron V. Cui Q. Wegner C. Antel J. Bruck W. (2008) Differentiation block of oligodendroglial progenitor cells as a cause for remyelination failure in chronic multiple sclerosis. Brain: A Journal of Neurology 131: 1749–1758. 18515322 10.1093/brain/awn096

[bibr25-1759091416668430] Lemke G. Lu Q. (2003) Macrophage regulation by Tyro 3 family receptors. Current Opinion in Immunology 15: 31–36. 12495730 10.1016/s0952-7915(02)00016-x

[bibr26-1759091416668430] Li R. Chen J. Hammonds G. Phillips H. Armanini M. Wood P. Mather J. P. (1996) Identification of Gas6 as a growth factor for human Schwann cells. The Journal of Neuroscience: The Official Journal of the Society for Neuroscience 16: 2012–2019. 8604045 10.1523/JNEUROSCI.16-06-02012.1996 PMC6578513

[bibr27-1759091416668430] Liu Y. Holdbrooks A. T. De Sarno P. Rowse A. L. Yanagisawa L. L. McFarland B. C. Qin H. (2014) Therapeutic efficacy of suppressing the Jak/STAT pathway in multiple models of experimental autoimmune encephalomyelitis. Journal of Immunology 192: 59–72. 10.4049/jimmunol.1301513 PMC3934829 24323580

[bibr28-1759091416668430] Livak K. J. Schmittgen T. D. (2001) Analysis of relative gene expression data using real-time quantitative PCR and the 2(-Delta Delta C(T)) Method. Methods 25: 402–408. 11846609 10.1006/meth.2001.1262

[bibr29-1759091416668430] Lu Q. Lemke G. (2001) Homeostatic regulation of the immune system by receptor tyrosine kinases of the Tyro 3 family. Science 293: 306–311. 11452127 10.1126/science.1061663

[bibr30-1759091416668430] Mi S. Lee X. Hu Y. Ji B. Shao Z. Yang W. Pepinsky R. B. (2011) Death receptor 6 negatively regulates oligodendrocyte survival, maturation and myelination. Nature Medicine 17: 816–821. 10.1038/nm.2373 21725297

[bibr31-1759091416668430] Miyamoto Y. Torii T. Takada S. Ohno N. Saitoh Y. Nakamura K. Yamauchi J. (2015) Involvement of the Tyro3 receptor and its intracellular partner Fyn signaling in Schwann cell myelination. Molecular Biology of the Cell 26: 3489–3503. 26224309 10.1091/mbc.E14-05-1020 PMC4591693

[bibr32-1759091416668430] Nobuta H. Ghiani C. A. Paez P. M. Spreuer V. Dong H. Korsak R. A. Waschek J. A. (2012) STAT3-mediated astrogliosis protects myelin development in neonatal brain injury. Annals of Neurology 72: 750–765. 22941903 10.1002/ana.23670 PMC3514566

[bibr33-1759091416668430] O’Bryan J. P. Fridell Y. W. Koski R. Varnum B. Liu E. T. (1995) The transforming receptor tyrosine kinase, Axl, is post-translationally regulated by proteolytic cleavage. The Journal of Biological Chemistry 270: 551–557. 7822279 10.1074/jbc.270.2.551

[bibr34-1759091416668430] Owshalimpur D. Kelley M. J. (1999) Genomic structure of the EPHA1 receptor tyrosine kinase gene. Molecular and Cellular Probes 13: 169–173. 10369740 10.1006/mcpr.1999.0228

[bibr35-1759091416668430] Pierce A. M. Keating A. K. (2014) TAM receptor tyrosine kinases: Expression, disease and oncogenesis in the central nervous system. Brain Research 1542: 206–220. 24184575 10.1016/j.brainres.2013.10.049 PMC4141466

[bibr36-1759091416668430] Prieto A. L. O’Dell S. Varnum B. Lai C. (2007) Localization and signaling of the receptor protein tyrosine kinase Tyro3 in cortical and hippocampal neurons. Neuroscience 150: 319–334. 17980494 10.1016/j.neuroscience.2007.09.047 PMC2231337

[bibr37-1759091416668430] Prieto A. L. Weber J. L. Lai C. (2000) Expression of the receptor protein-tyrosine kinases Tyro-3, Axl, and mer in the developing rat central nervous system. The Journal of Comparative Neurology 425: 295–314. 10954847

[bibr38-1759091416668430] Prieto A. L. Weber J. L. Tracy S. Heeb M. J. Lai C. (1999) Gas6, a ligand for the receptor protein-tyrosine kinase Tyro-3, is widely expressed in the central nervous system1. Brain Research 816: 646–661. 9878891 10.1016/s0006-8993(98)01159-7

[bibr39-1759091416668430] Qi Q. R. Yang Z. M. (2014) Regulation and function of signal transducer and activator of transcription 3. World Journal of Biological Chemistry 5: 231–239. 24921012 10.4331/wjbc.v5.i2.231 PMC4050116

[bibr40-1759091416668430] Ravindran J. Agrawal M. Gupta N. Rao P. V. (2011) Alteration of blood brain barrier permeability by T-2 toxin: Role of MMP-9 and inflammatory cytokines. Toxicology 280: 44–52. 21112371 10.1016/j.tox.2010.11.006

[bibr41-1759091416668430] Saxena A. Martin-Blondel G. Mars L. T. RSL. (2011) Role of CD8 T cell subsets in the pathogenesis of multiple sclerosis. FEBS Letters 585: 6. 10.1016/j.febslet.2011.08.047 21910991

[bibr42-1759091416668430] Scheller A. Kirchhoff F. (2009) Astrocyte: Identification Methods. In: Squire L. R. (ed.) Encyclopedia of neuroscience, Oxford, England: Academic Press, pp. 573–577.

[bibr43-1759091416668430] Schmittgen T. D. Livak K. J. (2008) Analyzing real-time PCR data by the comparative C(T) method. Nature Protocols 3: 1101–1108. 18546601 10.1038/nprot.2008.73

[bibr44-1759091416668430] Schneider C. A. Rasband W. S. Eliceiri K. W. (2012) NIH Image to ImageJ: 25 years of image analysis. Nature Methods 9: 671–675. 22930834 10.1038/nmeth.2089 PMC5554542

[bibr45-1759091416668430] Schulz N. T. Paulhiac C. I. Lee L. Zhou R. (1995) Isolation and expression analysis of tyro3, a murine growth factor receptor tyrosine kinase preferentially expressed in adult brain. Molecular Brain Research 28: 273–280. 7723626 10.1016/0169-328x(94)00216-2

[bibr46-1759091416668430] Seo J. H. Miyamoto N. Hayakawa K. Pham L. D. Maki T. Ayata C. Arai K. (2013) Oligodendrocyte precursors induce early blood-brain barrier opening after white matter injury. The Journal of Clinical Investigation 123: 782–786. 23281396 10.1172/JCI65863 PMC3561802

[bibr47-1759091416668430] Shankar S. L. O’Guin K. Cammer M. McMorris F. A. Stitt T. N. Basch R. S. Shafit-Zagardo B. (2003) The growth arrest-specific gene product Gas6 promotes the survival of human oligodendrocytes via a phosphatidylinositol 3-kinase-dependent pathway. The Journal of Neuroscience 23: 4208–4218. 12764109 10.1523/JNEUROSCI.23-10-04208.2003 PMC6741089

[bibr48-1759091416668430] Stenhoff J. Dahlback B. Hafizi S. (2004) Vitamin K-dependent Gas6 activates ERK kinase and stimulates growth of cardiac fibroblasts. Biochemical and Biophysical Research Communications 319: 871–878. 15184064 10.1016/j.bbrc.2004.05.070

[bibr49-1759091416668430] Stolt C. C. Schlierf A. Lommes P. Hillgartner S. Werner T. Kosian T. Wegner M. (2006) SoxD proteins influence multiple stages of oligodendrocyte development and modulate SoxE protein function. Developmental Cell 11: 697–709. 17084361 10.1016/j.devcel.2006.08.011

[bibr50-1759091416668430] Tsiperson V. Li X. Schwartz G. J. Raine C. S. Shafit-Zagardo B. (2010) GAS6 enhances repair following cuprizone-induced demyelination. PLoS One 5: e15748. 21203420 10.1371/journal.pone.0015748 PMC3009745

[bibr51-1759091416668430] van der Star B. J. Vogel D. Y. Kipp M. Puentes F. Baker D. Amor S. (2012) In vitro and in vivo models of multiple sclerosis. CNS & Neurological Disorders Drug Targets 11: 570–588. 22583443 10.2174/187152712801661284

[bibr52-1759091416668430] Vouri M. An Q. Birt M. Pilkington G. J. Hafizi S. (2015) Small molecule inhibition of Axl receptor tyrosine kinase potently suppresses multiple malignant properties of glioma cells. Oncotarget 6: 16183–16197. 25980499 10.18632/oncotarget.3952 PMC4599264

[bibr53-1759091416668430] Weinger J. G. Brosnan C. F. Loudig O. Goldberg M. F. Macian F. Arnett H. A. Shafit-Zagardo B. (2011) Loss of the receptor tyrosine kinase Axl leads to enhanced inflammation in the CNS and delayed removal of myelin debris during experimental autoimmune encephalomyelitis. Journal of Neuroinflammation 8: 49. 21569627 10.1186/1742-2094-8-49 PMC3121615

[bibr54-1759091416668430] Wolswijk G. (2002) Oligodendrocyte precursor cells in the demyelinated multiple sclerosis spinal cord. Brain: A Journal of Neurology 125: 338–349. 11844734 10.1093/brain/awf031

[bibr55-1759091416668430] Yanagita M. Arai H. Nakano T. Ohashi K. Mizuno K. Fukatsu A. Kita T. (2001) Gas6 induces mesangial cell proliferation via latent transcription factor STAT3. The Journal of Biological Chemistry 276: 42364–42369. 11546821 10.1074/jbc.M107488200

[bibr56-1759091416668430] Zhang H. Jarjour A. A. Boyd A. Williams A. (2011) Central nervous system remyelination in culture—A tool for multiple sclerosis research. Experimental Neurology 230: 138–148. 21515259 10.1016/j.expneurol.2011.04.009 PMC3117145

[bibr57-1759091416668430] Zheng M. Wei J. Tang Y. Yang C. Wei Y. Yin X. Liu Q. (2014) ApoE-deficient promotes blood-brain barrier disruption in experimental autoimmune encephalomyelitis via alteration of MMP-9. Journal of Molecular Neuroscience: MN 54: 282–290. 24788224 10.1007/s12031-014-0291-x

