# Supplementary material for: Gas6 Promotes Oligodendrogenesis and Myelination in the Adult Central Nervous System and After Lysolecithin-Induced Demyelination
Source: ASN Neuro. 2016 Sep 14;8(5):1759091416668430. doi: 10.1177/1759091416668430 (PMC5027908; doi:10.1177/1759091416668430)
Supplement: Supplementary material [file Supplementary_Tables_1-2.pdf]

Goudarzi *et al*. 2016

Supplementary Table 1 – List of primers used for qRT-PCR, with primer/probe IDs from IDT (Leuven, Belgium).

Primer	Primer ID
Mouse *Tyro3*	Mm.PT.56a.12621621
Mouse *Axl*	Mm.PT.56a.1150678.g
Mouse *MerTk*	Mm.PT.56a.28527132
Mouse *ProS1*	Mm.58.8481028
Mouse *Gas6*	Mm.PT.587992530
Mouse *GAPDH*	Mm99999915.g1
Mouse *CDC40*	Mm01189619.m1
Mouse *GFAP*	Mm.PT.58.6609337
Mouse *Epha1*	Mm.PT.58.7989514
Mouse *Mmp9*	Mm.PT.58.10100097
Mouse *MBP*	Mm.PT.58.28532164
Human *GAPDH*	Hs.PT.58.40035104
Human *Tyro3*	Hs.PT.56a.38778546
Human *Axl*	Hs.PT.56a.1942285
Human *Gas6*	Hs.PT.56a.21535693.g

Supplementary Table 2 – List of mouse MS-relevant genes for qPCR array (RT² Profiler™; Qiagen, Hilden, Germany).

Disease Aspect	Gene List
Myelination	*Cd9, Hexb, Ifng, Mal, Mbp, Ntf3, Plp1, Pmp22, Sod1, Tgfb1*
T-cell activation & signaling	*Apc, Cd28, Cd4, Egr1, Foxp3, Icam1, Ifng, Il10, Il18, Il1b, Il2ra* (CD25)*, Il6, Il6st* (gp130)*, Mapk1* (Erk2)*, Sod1, Tgfb1, Vcam1*
Adaptive immunity	*C1s1, Cd40* (Tnfrsf5)*, Foxp3, Icam1, Ifng, Il10, Il18, Il1b, Il6st* (gp130)*, Tgfb1, Vegfa*
Cytokines & chemokines	*Ccl12* (MCP-5, Scya12)*, Ccl3* (Mip-1a)*, Ccl5* (RANTES)*, Ccl7* (mcp3)*, Csf1* (Mcsf)*, Cxcl10* (INP10)*, Cxcl11* (Itac, Ip9)*, Cxcl9* (Mig)*, Fasl* (Tnfsf6)*, Ifng, Il10, Il18, Il1b, Il6, Lta* (Tnfb)*, Tgfb2, Tnf, Vegfa*.
Inflammation	*Ccl5* (RANTES)*, Ednra, Gpx1, Il6, Il6st* (gp130)*, Jak2, Tnfrsf1a* (Tnfr1)*, C1s1, Ccl12 (*MCP-5, Scya12)*, Ccl3 (Mip-1a), Ccl7 (mcp3), Ccr1, Ccr5, Cd40* (Tnfrsf5)*, Cd44, Cxcl10* (INP10)*, Cxcl11* (Itac, Ip9)*, Cxcl9* (Mig)*, Fn1, Hif1a, Il10, Il13, Il1b, Il2ra* (CD25)*, Itgb2, Nfkb1, Stat3, Tgfb1, Tnf, Tnfrsf1b*
Apoptosis	*Akt1s1* (PRAS40)*, Bax, Bcl2, Bcl2l1 (Bcl-XL), Erbb3, Gpx1, Jak2, Jun, Ntf3, Sod1, Tgfb2, Tnf, Il10, Mapk1 (Erk2), Tnfrsf1b, Il6st* (gp130)*, Hdac1, Raf1, Apc, App, Ccl12* (MCP-5, Scya12)*, Cd28, Fasl* (Tnfsf6)*, Ifng, Il1b, Il2ra* (CD25)*, Il6, Itgb2, Mmp9, Nfkb1, Tgfb1, Tnfrsf1a* (Tnfr1)*, Adm, Cd44, Lta (Tnfb), Mal, Vegfa*
Cell adhesion molecules	*Apc, App, Bcl2, Ccl12* (MCP-5, Scya12)*, Ccl5* (RANTES)*, Ccr1, Cd4, Cd44, Cd9, Csf1* (Mcsf)*, Cxcr3, Erbb3, Ezr, Fn1, Icam1, Il18, Il1b, Itgb2, Jak2, Mag, Map2k1* (Mek1)*, Tgfb1, Tgfb2, Tnf, Vcam1*
Cellular stress	*Adm, Bcl2, Gpx1, Hif1a, Ifng, Il1b, Jak2, Jun, Ptpn11, Sod1, Tgfb2, Tnf*
Receptors	*Ccr1, Ccr5, Cd28, Cd4, Cd40 (Tnfrsf5), Cd44, Cxcr3, Ednra, Epha1, H2-Eb1, Icam1, Il2ra* (CD25)*, Il6st* (gp130)*, Itgb2, Nr2f1, Tnfrsf1a* (Tnfr1)*, Tnfrsf1b*
Transcription factors	*Egr1, Foxp3, Hdac1, Hif1a, Jun, Myc, Nfkb1, Nr2f1, Stat3*
Other MS genes	*Aspa, Edc4, Gfap, Gnai2, Grb2, Jak1, Phgdh, Rangap1, Top1, Tubb4a, Ywhah*
