# Supplementary material for: Gas6 Promotes Oligodendrogenesis and Myelination in the Adult Central Nervous System and After Lysolecithin-Induced Demyelination
Source: ASN Neuro. 2016 Sep 14;8(5):1759091416668430. doi: 10.1177/1759091416668430 (PMC5027908; doi:10.1177/1759091416668430)
Supplement: Supplementary material [file Supplementary_Figures_1-3.pdf]

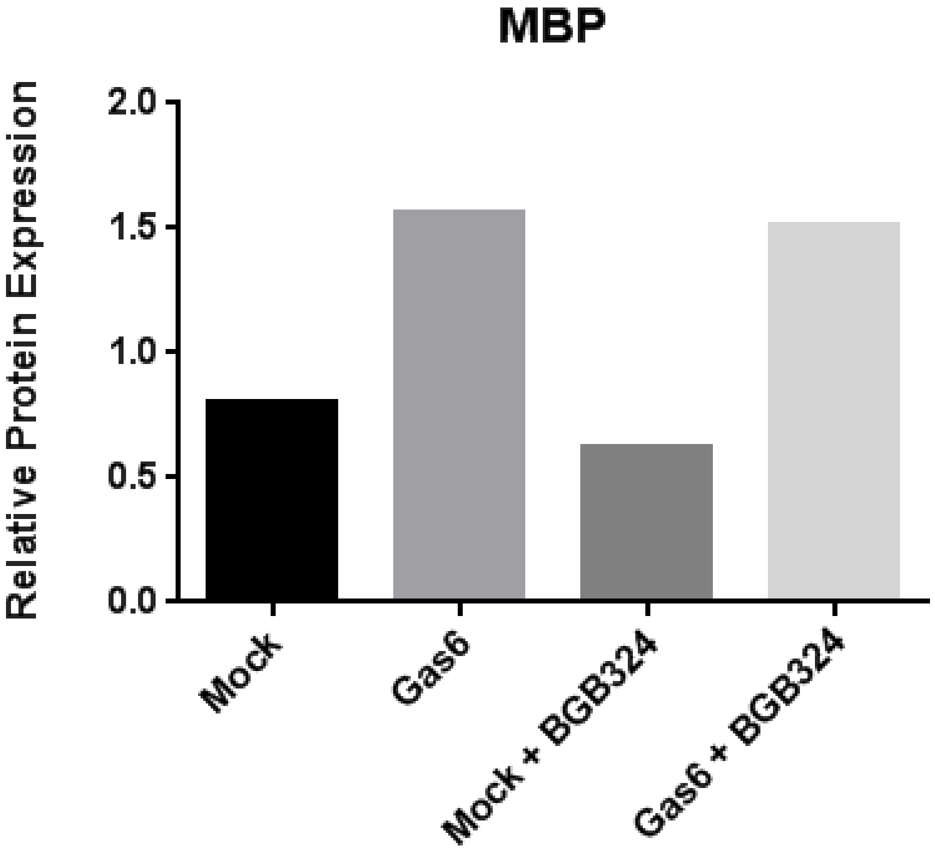

Supplementary Figure S1. The effect of a small molecule inhibitor of Axl, BGB324, on Gas6-induced MBP expression in optic nerve culture. BGB324 and co-incubation did not block the effect of Gas6 on MBP protein level. Results are densitometric quantification of Axl bands normalised to GAPDH protein bands, from a representative of two western blots.

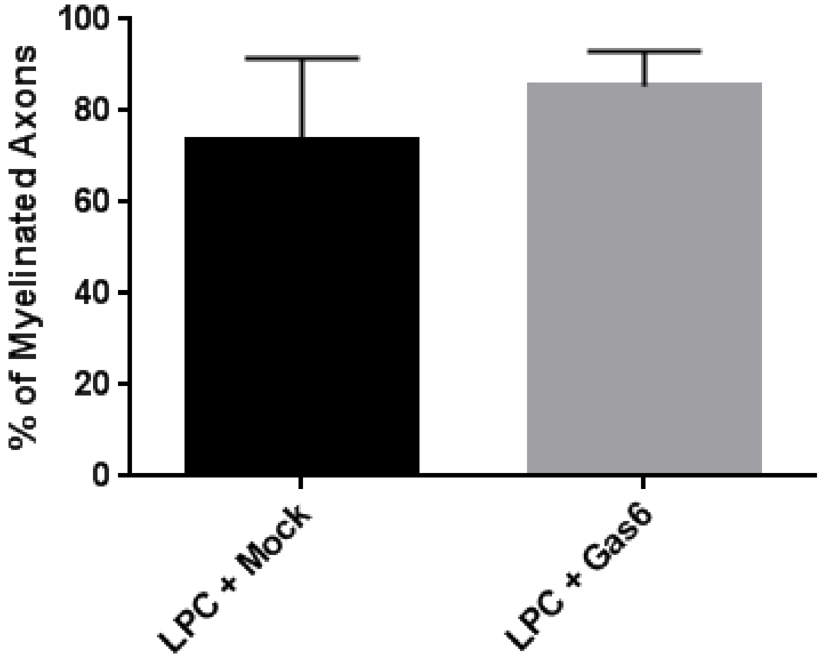

Supplementary Figure S2. The effect of Gas6 on remyelination of axons in cerebellar slice cultures. Gas6 was added to cerebellar slices 3 days following lysolecithin (LPC) withdrawal, and slices were incubated with mock and Gas6 medium for a further 3 days. Gas6 did not have a significant effect on remyelination (as described in *Methods*; n=3 fields of view per treatment).

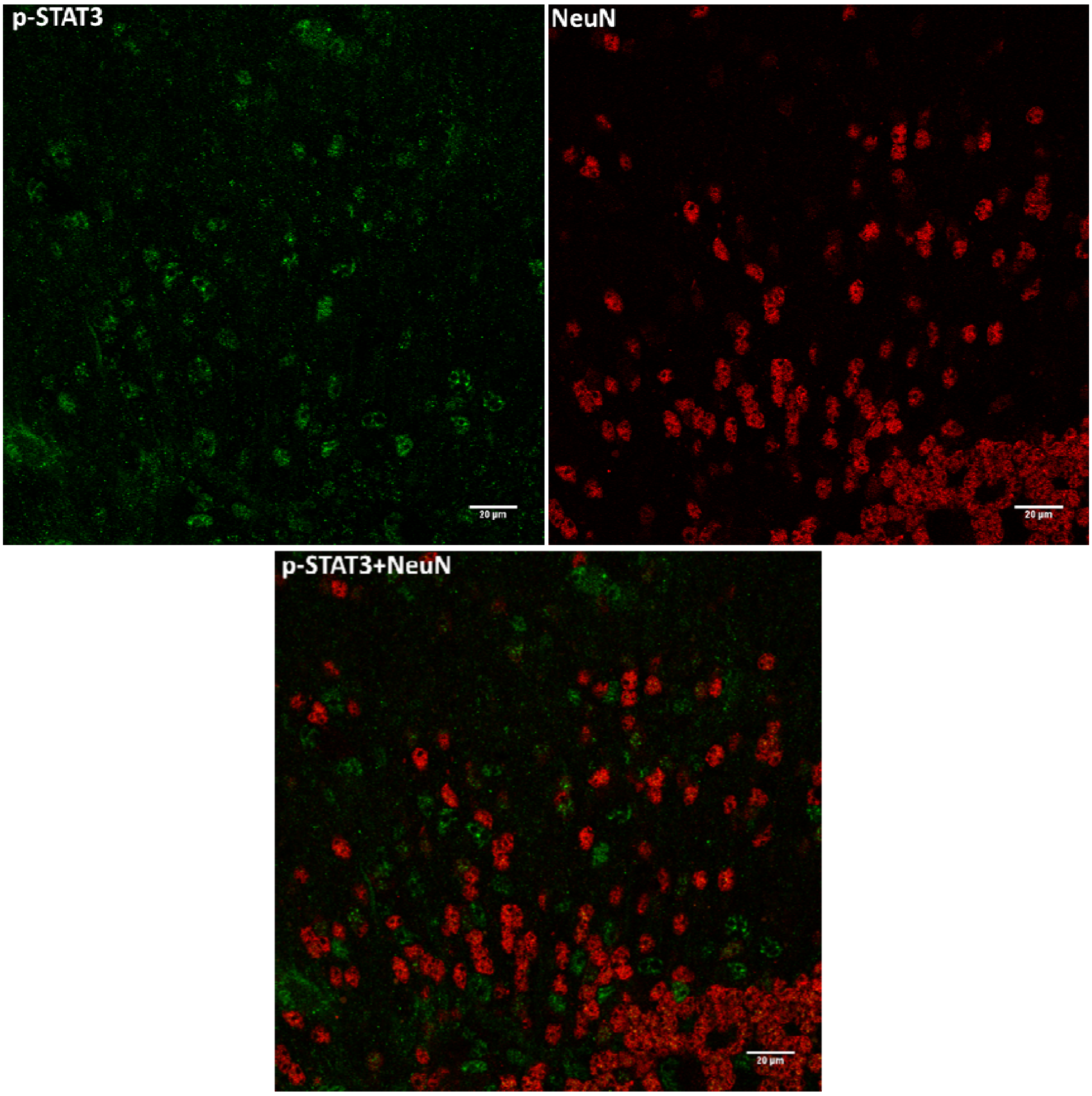

#

Supplementary Figure S3. Separate fluorescent channel images showing p-STAT3 (green) and NeuN (red) immunofluorescent staining (from Figure 6). The image shows that p-STAT3 and NeuN staining are mutually exclusive and identify distinct populations of cells.

#
